# Depressive Symptome in der Geriatrie – mehr als die Geriatrische Depressionsskala

**DOI:** 10.1007/s00391-025-02491-9

**Published:** 2025-08-26

**Authors:** Liv Silja Mertens, Katharina Geschke, Roland Hardt, Michael Mohr

**Affiliations:** 1 Abteilung für Geriatrie, Zentrum für Allgemeinmedizin und Geriatrie, Universitätsmedizin der Johannes Gutenberg-Universität Mainz, Mainz, Deutschland; 2 Klinik für Psychiatrie und Psychotherapie, Universitätsmedizin der Johannes Gutenberg-Universität Mainz, Mainz, Deutschland

**Keywords:** Ältere, Psychische Erkrankung, Prävalenz, GDS, Geschlecht, Elderly, Mental illness, Prevalence, GDS, Gender

## Abstract

**Hintergrund:**

Depressionen sind eine häufige, aber oft übersehene Problematik in der Versorgung älterer Menschen. Sie beeinträchtigen die Lebensqualität, verschlechtern somatische Erkrankungsverläufe und verursachen hohe Gesundheitskosten.

**Ziel:**

Untersuchung der Prävalenz depressiver Symptome bei akutgeriatrisch behandelten Patient*innen unter Berücksichtigung geschlechtsspezifischer Unterschiede sowie der Zusammenhänge mit kognitiven und funktionellen Einschränkungen.

**Material und Methoden:**

Retrospektive Analyse anonymisierter Daten von 345 Patient*innen, die 2019 in einer stationären Akutgeriatrie einer deutschen Universitätsmedizin behandelt wurden. Erfasst wurden GDS-15, MMST, Barthel-Index sowie dokumentierte Depressionsdiagnosen bei Aufnahme.

**Ergebnisse:**

Bei 286 Patient*innen lag eine vollständige Datengrundlage vor, 36,8 % zeigten eine auffällige depressive Symptomatik (GDS-15 > 5), nur bei 10,4 % war eine Depressionsdiagnose vorbekannt. Es ergaben sich keine signifikanten geschlechtsspezifischen Unterschiede. Der MMST korrelierte bei Frauen negativ mit der GDS-15; der Barthel-Index zeigte keinen Zusammenhang.

**Diskussion:**

Depressive Symptome im geriatrischen Setting sind häufig. Geschlechtsspezifische Unterschiede könnten im höheren Alter eine geringere Rolle spielen. Der fehlende Zusammenhang zwischen depressiven und funktionellen Symptomen, aber teils bestehende Zusammenhang mit kognitiven Einschränkungen verweist auf die diagnostische Herausforderung bei altersassoziierter Multimorbidität. Die Diskrepanz zwischen auffälliger depressiver Symptomatik und wenigen dokumentierten Depressionsdiagnosen weist auf mögliche Lücken in der vorstationären Erkennung und Erfassung depressiver Störungen hin.

## Abstract (en)

**Background:**

Depression is a common but often overlooked problem in the care of older people. It impairs the quality of life, worsens the course of somatic diseases and causes high healthcare costs.

**Objective:**

To investigate the prevalence of depressive symptoms in patients receiving acute geriatric treatment, taking gender-specific differences and correlations with cognitive and functional impairments into account.

**Material and methods:**

Retrospective analysis of anonymized data from 345 patients who were treated in an acute geriatric inpatient ward at a German university hospital in 2019. The geriatric depression scale (GDS-15), the mini-mental status test (MMST), Barthel index and documented depression diagnoses at admission were recorded.

**Results:**

Complete data were available for 286 patients and of these 36.8% showed noticeable depressive symptoms (GDS-15 > 5) but only 10.4% had a previous diagnosis of depression. There were no significant gender-specific differences. The MMST correlated negatively with the GDS-15 in women, while the Barthel Index showed no correlation.

**Discussion:**

Depressive symptoms are common in geriatric settings. Gender differences may play a lesser role in older age. The lack of correlation between depressive symptoms and functional impairments but the partial correlation with cognitive impairments, points to the diagnostic challenge of age-related multimorbidity. The discrepancy between noticeable depressive symptoms and few documented diagnoses of depression indicates possible gaps in the prehospital detection and recording of depressive disorders.

## Einleitung

Depressionen zählen zu den häufigsten psychischen Erkrankungen in der Allgemeinbevölkerung. Für Deutschland wurde im Jahr 2023 eine 12-Monats-Prävalenz von 16,7 % berichtet [12]. Auch bei älteren Menschen ab 60 Jahren ist die Erkrankung weit verbreitet: In dieser Altersgruppe lag die Prävalenz im selben Jahr zwischen 19,3 und 21,5 % [12]. Besonders deutlich tritt die Problematik bei stationär behandelten geriatrischen Patient*innen zutage. Internationale Studien berichten hier von Prävalenzen depressiver Symptome bis zu 37,0 %, was auf eine erhebliche Belastung in diesem Versorgungsbereich hinweist [5, 8, 13].

Studien zeigen, dass depressive Symptome im Alter häufig mit kognitiven Einschränkungen, körperlichen Komorbiditäten, aber auch chronischem Schmerz assoziiert sind [2, 16]. Dabei beeinflussen sich somatische und psychische Erkrankungen wechselseitig: Depressionen können den Verlauf körperlicher Erkrankungen ungünstig beeinflussen, den Abbau kognitiver Fähigkeiten beschleunigen und sind wiederum häufig Folge chronischer Belastungssituationen [2, 3]. Sie stellen eine relevante Herausforderung dar, da sie nicht nur die Lebensqualität der Betroffenen erheblich beeinträchtigen, sondern auch mit verlängerten Krankenhausaufenthalten und einer erhöhten Mortalität assoziiert sind [15, 17]. Dennoch bleiben depressive Symptome im klinischen Alltag älterer Patient*innen häufig unerkannt oder unzureichend behandelt [15, 18] – möglicherweise, weil sie unspezifisch verlaufen oder fälschlich als altersbedingte Veränderungen interpretiert werden.

In der stationären geriatrischen Versorgung bestehen besondere diagnostische und therapeutische Herausforderungen: Viele Patient*innen leiden unter multiplen körperlichen und kognitiven Einschränkungen, die mit depressiven Symptomen interagieren können [3, 4]. Studien aus dem Bereich der geriatrischen Rehabilitation zeigen, dass ausgeprägte depressive Symptome mit einem schlechteren funktionellen Behandlungserfolg einhergehen und die Wahrscheinlichkeit für erneute stationäre Aufnahmen erhöhen [14, 20].

Darüber hinaus wird zunehmend diskutiert, inwiefern geschlechtsspezifische Unterschiede auch im höheren Lebensalter eine Rolle bei Symptomatik, Diagnostik und Versorgung depressiver Störungen spielen [9]. Die aktuelle Studienlage belegt, dass Frauen in der Allgemeinbevölkerung häufiger von Depressionen betroffen sind als Männer [6]. Dieser Geschlechterunterschied zeigt sich jedoch nicht über alle Altersgruppen hinweg gleich stark [19]. Diese altersabhängige Dynamik legt nahe, dass sowohl biologische als auch psychosoziale Faktoren in unterschiedlichen Lebensphasen eine variable Rolle spielen könnten.

Trotz der hohen Relevanz depressiver Symptome im höheren Lebensalter liegt bislang nur eine begrenzte Datenlage zur spezifischen Prävalenz in der Akutgeriatrie vor. Insbesondere geschlechtsspezifische Unterschiede wurden in diesem Versorgungsbereich bislang kaum systematisch untersucht. Auch potenzielle Zusammenhänge zwischen depressiven Symptomen und funktionellen bzw. kognitiven Einschränkungen bleiben in der akutgeriatrischen Kohorte weitgehend unerforscht. Damit besteht eine Forschungslücke, die für eine differenzierte Diagnostik und Versorgung älterer Patient*innen von hoher Bedeutung ist.

Vor diesem Hintergrund wurde in der vorliegenden Studie die Prävalenz depressiver Symptome bei akutgeriatrisch behandelten Patient*innen untersucht. Dabei wurde ein besonderer Fokus auf geschlechtsspezifische Unterschiede gelegt. Weiterhin wurde analysiert, ob sich Zusammenhänge mit kognitiven (Mini-Mental-Status-Test; MMST) oder funktionellen Einschränkungen (Barthel-Index) zeigten, und es wurde erfasst, wie häufig bereits vorstationär eine Depressionsdiagnose bestand.

## Methodik

Grundlage der vorliegenden Studie ist die retrospektive Auswertung der ärztlichen Entlassungsbriefe von 345 Patient*innen, die 2019 in der Abteilung für Geriatrie der Universitätsmedizin Mainz behandelt wurden. Im Rahmen des standardisierten Aufnahmeassessments kamen folgende Screeninginstrumente zur Anwendung: die Geriatrische Depressionsskala (GDS-15) zur Erfassung depressiver Symptome, der MMST zur Einschätzung der kognitiven Einschränkungen sowie der Barthel-Index zur Bewertung der funktionellen Einschränkungen. Die GDS-15 konnte bei 288 Patient*innen ermittelt werden. Barthel-Index und MMST konnten bei 286 von diesen Patient*innen ermittelt werden. Nichterhobene Daten resultierten aus schlechtem Allgemeinzustand oder zu stark ausgeprägten kognitiven Einschränkungen. Zusätzlich wurde überprüft, ob in den vorliegenden Ärzt*innenbriefen oder Diagnoselisten der Hausärzt*innen bereits eine Depressionsdiagnose dokumentiert war.

### Geriatrische Depressionsskala

**Abb. 1:**
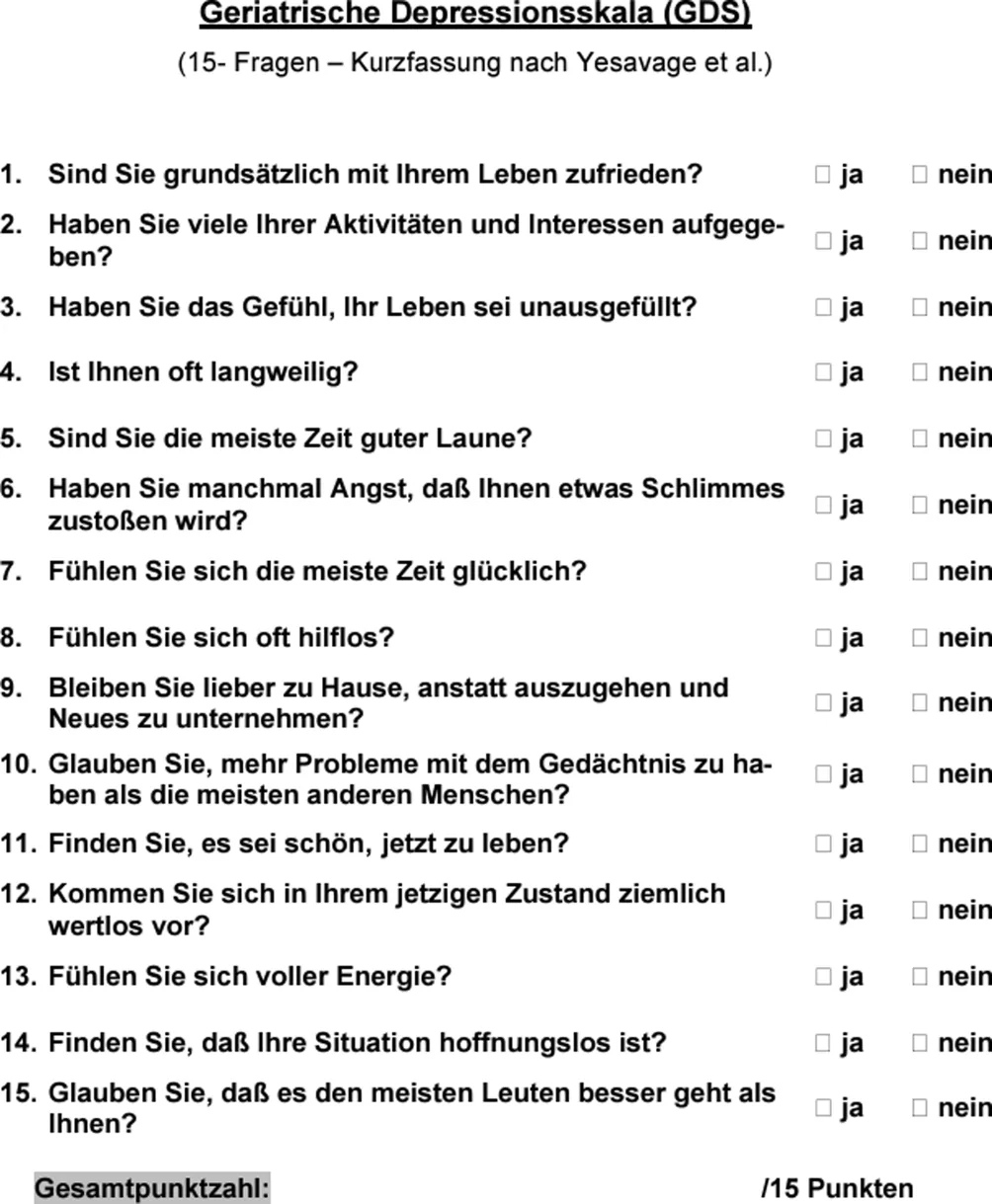
*Geriatische Depressionsskala (GDS-15) nach *Yesavage et al. [11]

Die GDS-15 wurde 1982 von Yesavage et al. entwickelt. Es handelt sich dabei um einen Fragebogen mit 15 Items, der die Bereiche *Stimmung, Antrieb, Zukunftsängste, Kognition und Selbstzweifel* abdeckt. 0 bis 5 Punkte gelten als unauffällig, 6 bis 10 Punkte als Hinweis auf eine leichte bis mäßige depressive Symptomatik, und ab 11 Punkten ist eine schwere depressive Symptomatik wahrscheinlich ([21]; siehe Abb. 1).

Die Skala eignet sich zur Selbsteinschätzung der depressiven Symptomatik, setzt aber eine gewisse Reflexionsfähigkeit voraus, weshalb ihre Aussagekraft bei fortgeschrittenen kognitiven Defiziten eingeschränkt ist [1]. Sie ersetzt keine umfassende Diagnostik bezüglich des Vorliegens einer Depressionsdiagnose. Bei einem Trennwert von 6 Punkten zwischen *keiner depressiven Symptomatik* und einer *leichten depressiven Symptomatik* erreicht die GDS-15 eine Sensitivität von 84,0 % und eine Spezifität von 88,9 %. Sie hat zudem eine hohe innere Konsistenz von 0,91 (Cronbachs α) [10].

### Barthel-Index

Der Barthel-Index ist ein Messinstrument zur Beurteilung der Alltagsfähigkeit in 10 Bereichen (Summenwert 0 bis 100 Punkte). Die Endsumme wird wie folgt interpretiert [10]:

- 0 bis 30 Punkte: weitgehend pflegeabhängig,
- 35 bis 80 Punkte: hilfsbedürftig,
- 85 bis 95 Punkte: punktuell hilfsbedürftig,
- 100 Punkte: komplette Selbstständigkeit.

Die Stärke des Barthel-Index liegt in der klaren Struktur und der guten Quantifizierbarkeit verschiedener Stufen von Pflegebedürftigkeit. Allerdings ist seine Aussagekraft begrenzt: Er erfasst ausschließlich bestimmte, standardisierte Aktivitäten des täglichen Lebens und lässt dabei andere relevante Alltagskompetenzen unberücksichtigt. So kann bei gleichem Barthel-Wert der tatsächliche Unterstützungsbedarf im Alltag individuell stark variieren. Trotz dieser Limitationen gilt der Barthel-Index als das am weitesten verbreitete Instrument zur Einschätzung der Alltagsfähigkeit.

### Mini-Mental-Status-Test

Der MMST ist ein Verfahren zur Erfassung der kognitiven Einschränkungen von Patient*innen. Er dient insbesondere als Screeninginstrument und ist für den klinischen Alltag gut geeignet, da er nur ca. 10 min dauert und einfach durchführbar ist. Die Sensitivität des Tests für eine Demenzdiagnose beträgt 87,0 %; die Spezifität beträgt 82,0 % [7].

Der MMST gliedert sich in 5 Bereiche: Orientierung, Merkfähigkeit, Aufmerksamkeit und Rechenfähigkeit, Erinnerungsfähigkeit, Sprache.

Es sind maximal 30 Punkte zu erreichen. Die Auswertung ist:

- MMST 27 bis 30 Punkte: keine kognitiven Einschränkungen,
- MMST 20 bis 26 Punkte: leichte kognitive Einschränkungen,
- MMST 10 bis 19 Punkte: moderate/mittelschwere kognitive Einschränkungen,
- MMST weniger als 10 Punkte: schwere kognitive Einschränkungen.

Trotz seiner weiten Verbreitung und guten Praktikabilität ist der MMST kein diagnostisches Instrument im engeren Sinne. Er liefert lediglich Hinweise auf kognitive Einschränkungen und ersetzt keine umfassende neuropsychologische Diagnostik [7]. Zudem können kognitive Einschränkungen vielfältige Ursachen haben – darunter auch reversible somatische Faktoren wie Infektionen, Stoffwechselstörungen oder Medikamentennebenwirkungen. Bei auffälligen Ergebnissen sollte daher immer eine weiterführende, differenzialdiagnostische Abklärung erfolgen.

### Definition und Erfassung depressiver Symptome

In der vorliegenden Studie wurde mittels GDS-15 das Vorliegen depressiver Symptome erfasst – eine vollständige gerontopsychiatrische Diagnostik erfolgte nicht. Daher wurde bewusst von „depressiven Symptomen“ gesprochen. In Bezug auf bereits dokumentierte Depressionsdiagnosen wurden sämtliche vorbestehende Depressionsdiagnosen gemäß ICD oder DSM berücksichtigt. Wenn keine formale Kodierung vorlag, wurde auch eine entsprechende Freitextbeschreibung (z. B. „Depression“) in die Auswertung einbezogen. Um ein möglichst vollständiges Bild der Versorgungssituation abzubilden, wurde Literatur mit verschiedenen diagnostischen Kriterien herangezogen, u. a. nach ICD-10 und DSM‑5.

### Statistik

Verwendet wurde die Statistik-Software SPSS in der Version 27. Es wurden Mittelwerte und Standardabweichungen (SD) angegeben. Getestet wurde auf Signifikanz mittels *t*-Test für unabhängige Stichproben sowie mittels Pearson-Chi-Quadrat-Test. Korrelationen wurden mittels Pearson-Korrelationskoeffizient bestimmt. Zusätzliche Risiken wurden mittels Odds Ratio berechnet. Für alle Tests wurde das Signifikanzniveau mit α = 0,05 gewählt.

## Ergebnisse

Von den 288 Patient*innen mit erfasster GDS-15 waren 67,0 % (*n* = 193) weiblich und 33,0 % (*n* = 95) männlich. Das mittlere Alter lag zum Erhebungszeitpunkt bei 83,7 Jahren (SD ± 6,6). Das Durchschnittsalter der Frauen betrug 84,0 Jahre (SD ± 6,5), das der Männer 83,1 Jahre (SD ± 6,8).

**Abb. 2:**
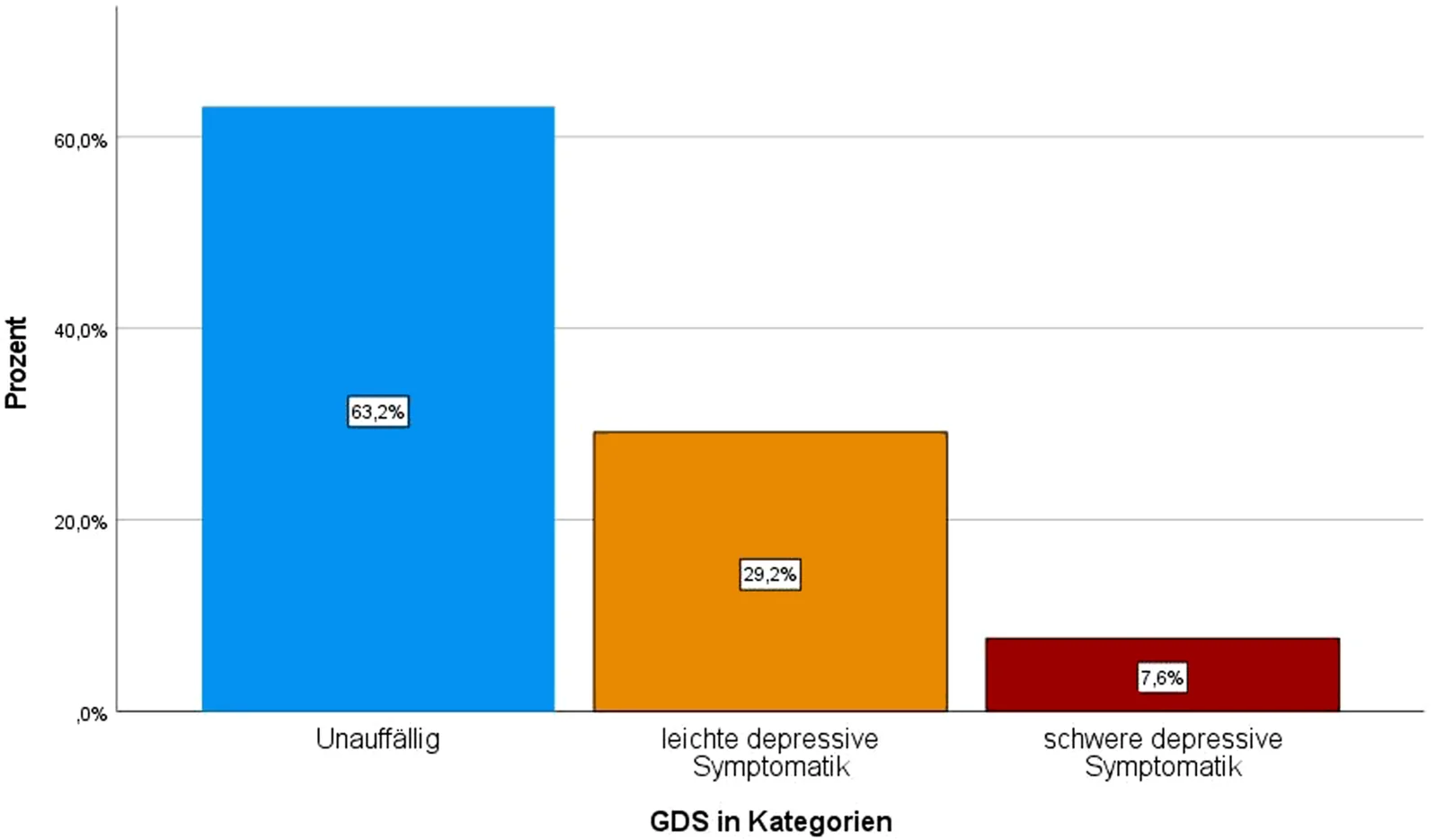
Verteilung der Patient*innen (in Prozent) nach Schweregrad der depressiven Symptomatik gemäß Geriatrische Depressionsskala (GDS-15) bei stationärer Aufnahme. (Eigene Grafik)

Der durchschnittliche GDS-15-Wert betrug 4,9 Punkte (SD ± 3,4). 63,2 % (*n* = 182) der Patient*innen hatten einen GDS-15-Wert von 0 bis 5 Punkten und zeigten somit keine Hinweise auf eine relevante depressive Symptomatik. 29,2 % (*n* = 84) lagen mit einem GDS-Wert zwischen 6 und 10 im Bereich einer leichten depressiven Symptomatik, während 7,6 % (*n* = 22) einen Wert über 10 Punkten aufwiesen, was auf eine schwere depressive Symptomatik hinweist (Abb. 2).

Der durchschnittliche GDS-15-Wert lag bei Frauen bei 5,1 Punkten (SD ± 3,5) und bei Männern bei 4,6 Punkten (SD ± 3,1) (T(286) = 1,07; *p* = 0,284).

**Abb. 3:**
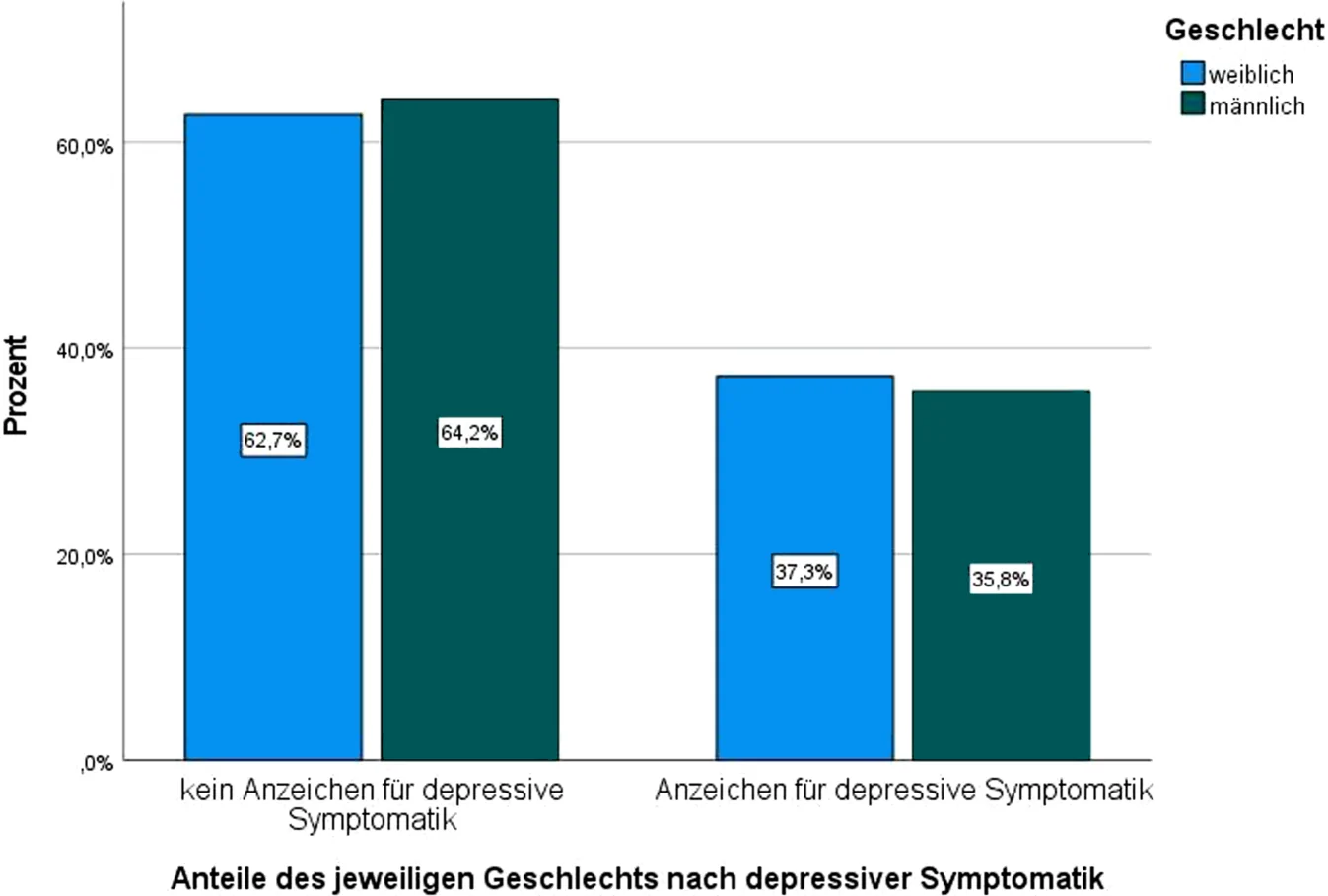
Anteil der Patient*innen mit und ohne Hinweise auf depressive Symptomatik (Geriatrische Depressionsskala (GDS-15), Cut-off >5 Punkte), bei stationärer Aufnahme, getrennt nach Geschlecht. (Eigene Grafik)

Hinweise auf eine depressive Symptomatik (GDS-15 > 5) zeigten 37,3 % (*n* = 72) der Frauen und 35,8 % (*n* = 34) der Männer (Abb. 3; Pearson-Chi-Quadrat: 0,063; *p* = 0,802).

Frauen mit Hinweisen auf eine depressive Symptomatik hatten in der GDS-15 im Schnitt 7,2 Punkte (SD ± 3,5), Männer mit Hinweisen auf eine depressive Symptomatik hatten in der GDS-15 im Durchschnitt 6,2 Punkte (SD ± 3,3) (T(155) = 1,74; *p* = 0,08).

Bei 11,4 % (*n* = 22) der Frauen und 8,4 % (*n* = 8) der Männer fand sich ein eine bestehende Depressionsdiagnose in den vorliegenden Vorbefunden (*p* = 0,437).

Von diesen 30 Patient*innen mit vorbekannter Depressionsdiagnose zeigten 70 % (*n* = 21) laut GDS-15 Anzeichen für eine depressive Symptomatik. Von diesen 21 Patient*innen mit vorbekannter Depressionsdiagnose und auffälligem GDS-15-Wert waren 76,2 % (*n* = 16) Frauen und 23,8 % (*n* = 5) Männer (Pearson-Chi-Quadrat: 0292; *p* = 0,589). 85,7 % (*n* = 18) der Patient*innen hatten dabei Anzeichen einer leichten depressiven Symptomatik, 14,3 % (*n* = 3) hatten Anzeichen einer schweren depressiven Symptomatik. Von den 18 Patient*innen mit einer leichten depressiven Symptomatik waren 77,8 % weiblich (*n* = 14) und 22,2 % männlich (*n* = 4). Von den 3 Patient*innen mit schwerer depressiver Symptomatik waren 66,7 % weiblich (*n* = 2) und 33,3 % männlich (*n* = 1).

Der durchschnittliche Barthel-Index aller Patient*innen betrug 46,1 Punkte (SD ± 21,9). Frauen erreichten im Durchschnitt 46,8 Punkte (SD ± 21,6), Männer 44,6 Punkte (SD ± 22,6) (T(284) = 0,777; *p* = 0,438). Zwischen dem Barthel-Index und dem GDS-15 zeigte sich keine signifikante Korrelation (Pearson: −0,090; *p* = 0,127). Es zeigte sich ebenso wenig ein Unterschied, betrachtete man nur die Frauen (Pearson: −0,90; *p* = 0,215), noch bei einzelner Betrachtung der Männer (Pearson: −0,103; *p* = 0,322).

Der durchschnittliche MMST-Wert lag bei 21,7 Punkten (SD ± 6,2) und damit im Bereich leichter kognitiver Einschränkungen. Frauen erreichten im Durchschnitt 21,6 Punkte (SD ± 6,1), Männer 21,8 Punkte (SD ± 6,2) (T(284) = −0,215; *p* = 0,830).

Es bestand eine signifikante negative Korrelation zwischen GDS-15 und MMST-Wert (Pearson: −0,132; *p* = 0,025), was bedeutet, dass mit zunehmender depressiver Symptomatik mehr kognitive Einschränkungen vorlagen und umgekehrt.

Betrachtet man ausschließlich die Männer, so zeigte sich kein signifikanter Zusammenhang (Pearson: −0,098; *p* = 0,347). Bei den Frauen hingegen war die negative Korrelation zwischen GDS-15 und MMST signifikant (Pearson: −0,147; *p* = 0,042), was auf einen möglichen geschlechtsspezifischen Zusammenhang zwischen depressiven Symptomen und kognitiven Einschränkungen hinweist.

## Diskussion

In der vorliegenden Untersuchung wiesen 36,8 % der akutgeriatrisch behandelten Patient*innen bei Aufnahme eine auffällige depressive Symptomatik gemäß GDS-15 auf. Diese Punktprävalenz lag deutlich über der in der Allgemeinbevölkerung berichteten 12-Monats-Prävalenz diagnostizierter Depressionen im höheren Lebensalter (z. B. 11,3 % laut Robert Koch-Institut [14] bzw. 13,8 % laut Statistischem Bundesamt [15]). Sie bewegte sich jedoch im Rahmen internationaler Studien mit stationär behandelten älteren Patient*innen, in denen Prävalenzen depressiver Symptome bis zu 37 % berichtet wurden [4, 13]. Dies verdeutlicht, dass depressive Symptome im stationären geriatrischen Kontext häufiger auftreten als im ambulanten Setting – möglicherweise begünstigt durch akute somatische Belastungen, funktionelle Einbußen oder psychosoziale Faktoren wie Hospitalisierung und Verlust von Autonomie.

Es zeigten sich in unserem Kollektiv keine signifikanten geschlechtsspezifischen Unterschiede in der Ausprägung depressiver Symptome. Dies widerspricht den Befunden aus der Allgemeinbevölkerung, in der Frauen deutlich häufiger von Depressionen betroffen sind [14]. Möglicherweise gleichen sich geschlechtsspezifische Unterschiede im höheren Lebensalter durch altersbedingte Veränderungen der Symptomatik, hormonelle Faktoren oder Unterschiede in der Symptomwahrnehmung aus. Eine weitere Erklärung könnte in der diagnostischen Untererfassung in der Allgemeinbevölkerung bei Männern liegen – insbesondere, wenn affektive Symptome weniger berichtet oder anders ausgedrückt werden.

Der Mini-Mental-Status-Test (MMST) korrelierte signifikant negativ mit dem GDS-15; nur wenn ausschließlich Männer betrachtet wurden, zeigte sich dieser Zusammenhang nicht. Dass Depressionen und kognitive Einschränkungen im Alter häufig gemeinsam auftreten, ist aus der Literatur bekannt [2], insbesondere im Kontext der diagnostischen Überschneidung mit demenziellen Syndromen. Die Differenzierung zwischen Depression und Demenz gestaltet sich im höheren Lebensalter häufig schwierig, da sich beide Erkrankungen symptomatisch überschneiden – beispielsweise im Bereich der Antriebsminderung, Konzentrationsstörung und Einschränkungen der Alltagskompetenz. Der hier beobachtete geschlechtsspezifische Unterschied bedarf weiterer Untersuchung, insbesondere mit Blick auf Unterschiede in Symptomwahrnehmung, -äußerung und Diagnosestellung.

Hingegen konnte in unserem Kollektiv kein signifikanter Zusammenhang zwischen dem Barthel-Index und dem GDS-15 festgestellt werden. Dies könnte weniger ein Hinweis auf das generelle Fehlen einer Assoziation sein als vielmehr auf den spezifischen Kontext unserer Untersuchung: Bei akutgeriatrisch behandelten Patient*innen mit akuten somatischen Erkrankungen (z. B. kardiale Dekompensation, Infektionen) sind sowohl die Alltagsfunktionalität als auch affektive Parameter häufig vorübergehend beeinträchtigt. Funktionelle Einschränkungen und depressive Symptome können in diesem Setting situativ verstärkt auftreten, ohne zwingend eine manifeste Depression oder dauerhafte Pflegebedürftigkeit anzuzeigen.

Auffällig ist zudem die Diskrepanz zwischen den GDS-15-Ergebnissen und den dokumentierten Depressionsdiagnosen bei Aufnahme: Obwohl 36,8 % eine auffällige depressive Symptomatik zeigten, lag nur bei 10,4 % eine dokumentierte Depressionsdiagnose bei Aufnahme vor. Auch hierbei zeigte sich kein signifikanter Unterschied zwischen den Geschlechtern, was darauf hindeuten könnte, dass geschlechtsspezifische Unterschiede in der Erkennung oder Diagnosestellung im höheren Lebensalter weniger stark ausgeprägt sind als in jüngeren Altersgruppen. Gleichzeitig wirft dieses Missverhältnis Fragen nach der diagnostischen Sensitivität im vorstationären Versorgungskontext auf: Möglicherweise werden depressive Symptome – unabhängig vom Geschlecht – bei geriatrischen Patient*innen häufig nicht als solche erkannt oder adäquat dokumentiert. Neben altersbedingten Besonderheiten in der Symptompräsentation könnte auch der Umstand eine Rolle spielen, dass bei der Aufnahme häufig keine vollständige medizinische Historie vorliegt und sich das Einholen von Vorbefunden primär auf somatische Diagnosen konzentriert.

Die vorliegenden Ergebnisse müssen unter Berücksichtigung mehrerer Limitationen interpretiert werden. Erstens bezieht sich die Erhebung auf ein spezifisches Patient*innenkollektiv in einer akutgeriatrischen Station, was die Generalisierbarkeit auf andere Versorgungskontexte einschränkt. Zweitens handelt es sich um eine Querschnitterhebung zum Zeitpunkt der stationären Aufnahme. Temporale Verläufe wurden nicht erfasst. Eine zusätzliche Erhebung am Ende des stationären Aufenthalts könnte hier helfen, besser zwischen akutsituativen Reaktionen und behandlungsbedürftigen depressiven Störungen zu unterscheiden. Drittens erfolgte die Erfassung bereits bekannter Depressionsdiagnosen ausschließlich anhand vorhandener Vorbefunde und ärztlicher Dokumentation bei Aufnahme. Nichtdokumentierte Diagnosen könnten zu einer systematischen Unterschätzung der Prävalenz führen.

Schlussendlich verdeutlichen unsere Ergebnisse den anhaltenden Handlungsbedarf in der Diagnostik und Versorgung depressiver Symptome im geriatrischen Setting. Sie unterstreichen die hohe Prävalenz depressiver Symptome im akutgeriatrischen Patient*innenkollektiv und zeigen zugleich, dass Männer und Frauen in dieser Kohorte keine signifikanten Unterschiede in der Ausprägung depressiver Symptome zeigen. Auch funktionelle Einschränkungen, gemessen am Barthel-Index, zeigten keinen Zusammenhang mit depressiven Symptomen. Es bestand eine signifikante Korrelation mit dem kognitiven Status (MMST) und depressiven Symptomen, betrachtete man jedoch ausschließlich die männlichen Patient*innen war dieses Ergebnis nicht signifikant. Diese Befunde sollten in weiteren Studien überprüft und vertieft werden, um geschlechtsspezifische Unterschiede und mögliche Interaktionen mit kognitiven und funktionellen Parametern besser zu verstehen. Perspektivisch bedarf es einer stärkeren Beachtung depressiver Symptome im geriatrischen Alltag. Bei auffälligen Screeningergebnissen sollte konsequent eine weiterführende, differenzierte Diagnostik eingeleitet werden, um eine angemessene Versorgung sicherzustellen.

## Fazit für die Praxis

Depressive Symptome in der Akutgeriatrie sind häufig, auch wenn nur selten eine Depressionsdiagnose laut Vorbefunden vorliegt. Männer und Frauen scheinen ähnlich stark betroffen zu sein. Zudem zeigten sich weder bei der funktionellen Selbstständigkeit (Barthel-Index) noch bei der kognitiven Leistungsfähigkeit (MMST) eindeutige geschlechtsspezifische Unterschiede.

Bei einem auffälligen Screeningbefund sollte konsequent eine weiterführende gerontopsychiatrische Diagnostik eingeleitet werden.

## Data Availability

Die in der aktuellen Studie verwendeten und/oder analysierten Datensätze sind auf begründete Anfrage beim entsprechenden Autor erhältlich.
